# The rationale for removing typification from virus taxonomy

**DOI:** 10.1007/s00705-025-06513-0

**Published:** 2026-01-07

**Authors:** Stuart G. Siddell

**Affiliations:** School of Cellular and Molecular Medicine, University of Bristol, Bristol, UK

## Abstract

Typification, the linkage of a name-bearing “type” to a taxon and the description and deposition of a corresponding type specimen, has been a principle of biological taxonomy for over two centuries. It was introduced to promote nomenclatural uniformity and stability. Until recently, a modified form of typification was also written in the rules governing the taxonomy of viruses. However, in 2021, the International Committee on Taxonomy of Viruses abolished all type species and removed the requirement to designate a type species when a new genus is created. In this article, I briefly review the history of typification, explain its purpose and evaluate its relevance to current virological practice.

## A brief history and the purpose of typification

The complex history of the term “type” in taxonomy is best left to the philosophers and historians of biology [1, 2]. In this short article, I am concerned only with the present use of the word “type” when referring to either *the name-bearing-type species of a genus*, i.e., the fixed nomenclatural element of a genus taxon, or the *name-bearing type specimen*, which is a physical entity (or a representation of that physical entity) from which the character(s) of a type species are determined. I am not concerned here with name-bearing types for taxa above the genus rank.

It is important to understand that typification is a nomenclatural concept. Classification is a different process that is concerned with how the boundaries of species taxa are determined and how species taxa are distinguished from one another by their diagnostic characters. There is no requirement for the type species (and its type specimen) to be typical of the taxon it represents. In other words, “a type specimen fixes the reference of a taxon name without defining it” [3]. The concept of typification gained traction in the mid-19th Century and was then maintained through the 20th Century. The earliest set of rules that explicitly propose typification was published by HE Strickland, an English ornithologist, in 1843 [4].

The main argument used to support typification is that it creates “anchor points” for nomenclatural uniformity and stability, i.e., avoiding ambiguity such as alternative names for the same taxon (synonymy), or the same name for alternative taxa (homonymy). The potential sources of these ambiguities lie not only in simple misidentification or procedural errors but also in changes that arise from taxonomic progress. For example, taxonomic species can be “split” or “lumped”, so that the boundaries of taxa are revised, and new taxa are created or abolished. With the linkage of a type species name to a specimen, there should be at least no ambiguity about what that name refers to. It may be that the description of the taxon changes (for example, by the addition of more, or different, diagnostic characters), but any disagreements can always be settled by referring back to the specimen.

Clearly, nomenclatural stability is a worthy goal; ambiguities are bad for taxonomy and detrimental in areas such as communication, teaching, medicine, pharmaceuticals, and regulatory affairs. But, as discussed below, stability should not be achieved at the cost of taxonomic stagnation. The provisions of nomenclature codes are a “means to an end” and not “an end in itself” [5]. As new knowledge becomes available (for example, more rigorous diagnostic characters) and, as the taxonomic process is increasingly centralized and computerized, the need for typification and specimen deposition will diminish.

## Typification rules in animal, plant and prokaryotic codes

The International Code of Zoological Nomenclature (ICZN) [6], the International Code of Nomenclature for algae, fungi, and plants (ICNAFP) [7] and the International Code of Nomenclature of Prokaryotes (ICNP) [8] all embody, in various forms, the principle of typification, i.e., the designation of a type species and the deposition of a type specimen. In all 3 codes, there are also requirements to designate types for families (ICZN and ICNAFP) or families, orders, classes, and phyla (ICNP)

The ICZN Code defines the type species as “the nominal species that is the name-bearing type of a nominal genus or subgenus”. The type specimen is “An animal (colonies thereof or parts thereof) and fossils, both of which may be represented by an illustration or description” ([6], Article 72). It is important to note that when a description is used (and this may be, for example, a DNA sequence determined from the tissues of an animal), it is the animal (or its tissue) that remains the type specimen. In other words, the DNA sequence alone does not constitute a type specimen.

The ICNAFP Code has a similar position regarding typification, although the botanical type species has no formal standing. The type specimen must be a preserved physical sample (or, in some special cases, an illustration thereof) deposited in an appropriate institution ([7] Article 8). The term physical specimen is understood to exclude a DNA sequence. It should be noted that this position is currently being challenged, especially in the area of fungal taxonomy, and may well be amended [9, 10].

The ICNP Code parallels the animal and plant codes. The definition of the type specimen (also refered to as a strain) is, however, more restrictive because from 2001 onwards, descriptions, non-viable (preserved) specimens, or illustrations are no longer allowed as type specimens ([8], Section 4). Proposals to extend the definition of the prokaryote type specimen have also been discussed at length [11, 12], but have not yet been agreed upon.

One significant consequence of the rules described above is that so-called “dark taxa”, namely taxa created on the basis of genome sequences derived from the metagenomic analysis of environmental samples, remain without a formal name in organismal taxonomy. This has now become a central debate and has even led to an alternative, sequence-based animal taxonomy called SeqCode [13]. There are attempts to unite the ICZN code and SeqCode but they have not yet reached a conclusion [14].

## The virus code

The International Committee on Taxonomy of Viruses (ICTV) is uniquely mandated to develop an internationally agreed-upon taxonomy for viruses, to establish internationally agreed-upon names for virus taxa, and to maintain an official index of approved names for virus taxa. Its rules regarding the nomenclature of virus taxon names are published in the International Code of Virus Classification and Nomenclature (ICVCN) (https://ictv.global/about/code). The ICVCN also stipulates that the nomenclature of virus taxa is independent of other biological nomenclatures.

The first mention of typification in virus taxonomy is found, rather hidden from view, in the Minutes of the 1966 International Congress of Microbiology held in Moscow in 1966, when the ICTV (then called the International Committee on Nomenclature of Viruses, ICNV) was founded [15].

“A species shall be selected to typify each genus. This type species shall be selected as being the most fully defined by published work (e.g. by serotype and other criteria), and should desirably be represented in a Type Culture Collection and held and maintained in replicate in 4 or 5 different countries” [15].

However, this rule remained as a proposal until the 4th ICTV Report of 1982 [16], which then states explicitly: “Rule 22-Approval of a new family must be linked to the approval of a type genus; approval of a new genus must be linked to the approval of a type species”. However, there was no longer any mention of the need to deposit an archived type specimen. Also, in practice, the requirement to designate a type genus for each family was never enforced by the ICTV. Paradoxically, at the time these stipulations were made, the ICTV had not officially accepted the concept of a virus species, nor had a definition of a virus species been formulated, both of which happened only in 1991[17]. This, however, did not stop the ICTV designating “type species” and “type members” from the outset [18].

This situation remained until 2021 when the ICTV membership voted to abolish all type species and remove the requirement for a type species as a designated nomenclatural type for each genus [19]. At that time, the rationale provided for this decision was simply that the designation of a “type species no longer serves a clear and useful purpose”.

## 21st century virus taxonomy and typification

There are several reasons to justify the removal of the typification principle in virus taxonomy. First, the taxonomy of viruses is developed and overseen by the ICTV, which scrutinizes, approves and ratifies taxonomic proposals on an annual basis. Virus taxonomy is developed from within the virology community, but it is regulated by a central body [20]. This means that the naming of taxa can be closely monitored. Second, Rule 3.9 of the ICVCN code says the “rule of priority” in naming taxa shall not be observed. Therefore, it is not possible to invalidate a name in current use by claiming priority for an older name. Third, in contrast to other codes, the virus code has no absolute requirement for a formal taxonomic description. The creation of a new species taxon requires only that “ Each new species taxon must be assigned to a genus (whether existing or novel) and proposals to create a new species taxon need to state the species demarcation criteria established for the genus, and show that the candidate species meets these requirements” (https://ictv.global/taxonomy/templates). There is no requirement to deposit a physical type specimen.

A spreadsheet of approved virus species names, the Master Species List (MSL), is published by the ICTV annually (https://ictv.global/msl) and it is easy to check that taxon names are unique. Also, the ICTV publishes the updated online Report (https://ictv.global/report/genome) that includes chapters with extensive information on virion structure, genome structure, replication, biology, phylogeny, and detailed multiple sequence alignments of “hallmark” genes for individual families. This resource is important in facilitating the preparation of robust and accurate taxonomic proposals. With this framework in place, the dangers of oversight or error are significantly reduced and, altogether, it provides a well-regulated infrastructure with the flexibility needed to respond to changes in a rapidly developing taxonomy.

It should be noted that although the ICTV has removed the requirement to designate a type species for each genus, it still encourages practices that are designed to provide a conceptual bridge between related old and new genera. For example, the genus *Flavivirus* has recently been renamed as *Orthoflavivirus* (from Greek “*orthos*” straight or true), to clarify the difference between the family and genus names. There are now 25 genera with this prefix. It could be argued that changing taxon names, as described above, or the recently introduced change to binomial virus species names [21] makes the tracking of classification histories more difficult. However, the ICTV provides a Find the Species tool (https://ictv.global/search/find_the_species) that facilitates the searching of both current and historical versions of ICTV databases as well as INSDC entries using virus names, virus taxon names and some disease names.

## Type specimens and exemplar sequences

Although virus nomenclature and virus classification are separate processes, they are closely linked. In the last two decades virus classification has been revolutionized by the introduction of rapid, inexpensive, high-throughput sequencing. In 1999, there were about 1500 recognized virus species taxa. As of 2025, there are over 16000 species taxa. A large proportion of this increase is due to “dark taxa”, viruses that are only known from their genome sequence. Typically, these viruses cannot be directly associated with a phenotype or host. Nevertheless, thanks to the scale of metagenomic characterization, the viruses of “dark taxa” constitute most of the known diversity in the virosphere and they are now recognized as *bona-fide* viruses by the ICTV [22].

The ICTV requires the deposition and publication of a complete (or, at least, coding-complete) genome sequence of an exemplar virus when a new species taxon is created (https://ictv.global/about/code). If the standards of sequence quality, assembly, completeness, and annotation are observed [23], a genome sequence is a character set that is accurate, high-resolution, and can be stored indefinitely. If necessary, it is even possible to use reverse genetic approaches to generate an infectious virus, based on the genome sequence alone [24, 25]. In most cases, this exemplar sequence is the basis of classification. This usually takes the form of a phylogenetic comparison (monophyly is an ICTV requirement for classification of a taxon) and a metric of genetic relatedness that defines the boundaries between inclusion of the virus in an existing species taxon, or the creation of a new species taxon. Phylogenetic comparisons should include only orthologous genes and, if possible, multiple sequences obtained from independent studies.

Exemplar sequences are listed for virus species in the Virus Metadata Resource (https://ictv.global/vmr), a spreadsheet that is published by the ICTV, together with each MSL release. The exemplar virus genome sequences are chosen by the authors of taxonomic proposals, and they can be changed, for example if the original exemplar genome sequence was incomplete or aberrant or was derived from a variant that was poorly characterised. As with a type specimen, the genome sequence of an exemplar virus may have features that are not typical of the species, but the defining characters will be shared with other taxon members. Whether or not the genome sequence of an exemplar virus can be considered as a proxy type specimen is a matter of debate, but it does not have any relevance to the naming of species taxa.

## Conclusion

Although the concept of a type species was originally part of virus taxonomy, this concept has been abandoned because virus taxonomy is decided by the ICTV rather than through the priority of publication. The identity and scope of genera (and higher taxa) can be discovered by reference to an annually updated list of all taxa (the MSL) rather than by reference to types. Similarly, physical “type specimens” are not needed for virus species, since the complete genome sequence of an exemplar virus strain is required and acts as a proxy for a physical specimen. As genome sequencing is applied more extensively to cellular life, it is possible that the animal, plant and prokaryotic taxonomic codes will also move away from rank typification and physical type specimens. Genome-based classification opens the way to a more informed view of the extent and diversity of the virosphere. The current view of the ICTV, as embodied in the ICVCN, is that the advantages of using this flexible approach outweigh any potential risks of removing the requirement for type species and type specimens.
